# Dr. Philo G. Hubbell

**Published:** 1915-06

**Authors:** 


**Dr. Philo G. Hubbell,** (not listed in State Directory), Eclectic of Cincinnati, 1871, died in Syracuse, April 12, aged 72.

